# Key Milestones Contributing to the Understanding of the Mechanisms Underlying Fibromyalgia

**DOI:** 10.3390/biomedicines8070223

**Published:** 2020-07-17

**Authors:** Geoffrey Littlejohn, Emma Guymer

**Affiliations:** 1 Departments of Medicine, Monash University, Melbourne 3168, Australia; emma.guymer@monash.edu; 2 Departments of Rheumatology, Monash Health, Melbourne 3168, Australia

**Keywords:** fibromyalgia, 1990 ACR criteria, mechanisms, central sensitization

## Abstract

The promulgation of the American College of Rheumatology (ACR) 1990 criteria for fibromyalgia (FM) classification has significantly contributed to an era of increased research into mechanisms that underlie the disorder. The previous emphasis on putative peripheral nociceptive mechanisms has advanced to identifying of changes in central neural networks that modulate pain and other sensory processes. The influences of psychosocial factors on the dynamic and complex neurobiological mechanisms involved in the fibromyalgia clinical phenotype are now better defined. This review highlights key milestones that have directed knowledge concerning the fundamental mechanisms contributing to fibromyalgia.

The clinical features that characterise the phenotype designated by fibromyalgia (FM) have long been described in both general and medical literature. However, the promulgation of the American College of Rheumatology (ACR) 1990 classification criteria for fibromyalgia [1] triggered a marked increase in focused research into clinical, social, and mechanistic aspects of the disorder. The criteria acted as a watershed for better understanding and management of this highly impactful and common disorder. We reviewed selected observations on mechanisms deemed to be important in fibromyalgia, with an emphasis on neurophysiological processes, but with recognition of significant input from social and psychological factors (Table 1).

## 1. Describing Fibromyalgia

Fibromyalgia is characterised by a distinctive collection of contributing features that typically include widespread pain and tenderness, muscle tightness, peripheral dysesthesia, soft tissue swelling, emotional distress, poor quality sleep, fatigue, and cognitive dysfunction [2]. These features are described in ancient writings and appear in medical descriptions from the 19th century [3,4], reflecting the ubiquitous presence of this condition.

Fibromyalgia, under many different names, was more clearly defined over 100 years ago, but it was not until the 1970s that Smythe and Moldofsky described the crucial clinical characteristics of the disorder [5]. They focused on the clinical features of widespread pain and tenderness; they noted that particular sites in the body (known as “tender points”) were predictably more sensitive to palpation in those with fibromyalgia than in healthy controls. They also recognised several central characteristics (in contrast to peripheral) such as fatigue, poor sleep, and emotional distress. Others extended these observations and identified significant associations with conditions such as irritable bowel syndrome, irritable bladder syndrome, and migraine, among other disorders [6,7].

## 2. Evolution of Classification and Diagnostic Criteria

Classification criteria emerged in the 1970s, focusing on a mix of defining clinical features that included widespread pain, tenderness, and other symptoms [5,6]. The 1990 ACR classification criteria were a more robust refinement of previous criteria and better defined a fibromyalgia patient for purposes of research [1]. Later criteria focused on patients self-reporting their key symptoms; furthermore, the criteria recognised that the symptoms of fibromyalgia exist on a spectrum, allowing for a better understanding of the fluctuations in these symptoms, along with responses to therapies [8,9,10,11]. Other criteria were explored with different characteristics [12,13]. In the 2020s, newer criteria explore combinations of the self-reporting of key fibromyalgia-related clinical features, such as fatigue, insomnia, and bedside clinical measures of central sensitization, such as slowly repeated evoked pain responses [14]. These post-1990 ACR criteria have further enhanced the understanding of fibromyalgia’s key elements by both healthcare workers and patients.

**Figure 1:**
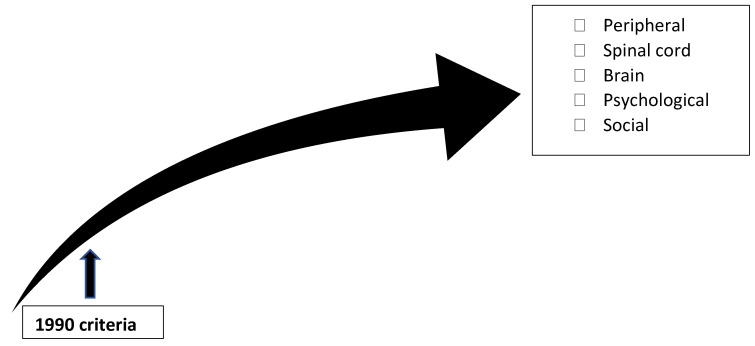
The 1990 ACR criteria accelerated and broadened the curve of knowledge of mechanisms contributing to fibromyalgia in many overlapping domains.

The 1990 ACR criteria are seen as a lift-off point for ongoing validated classification and diagnostic criteria, which can be used in different circumstances ranging from epidemiological to neurobiological explorations, in patients deemed to have fibromyalgia. Importantly, the criteria promulgated the change in name from the previous term, fibrositis, to fibromyalgia, one that had been suggested by others earlier [15]. This name change reflected a transformation in thinking of the underlying mechanisms contributing to fibromyalgia (Figure 1).

## 3. Exploration of Peripheral Muscle Mechanisms was Prominent Prior to the 1990 ACR Criteria

Key clinical features of fibromyalgia, such as pain, tenderness, and stiffness, are predominantly present in muscle and joint regions. Due to this, peripheral nociceptive causes for the condition have long been sought. Early concepts of mechanisms contributing to these features included muscle inflammation, fascia inflammation, or both [16,17]. Histomorphometry assessments of tissues taken from regions of pain in patients deemed to have fibromyalgia were initially thought to show soft-tissue inflammatory change [18]; this was part of the reason that the term “fibrositis” continued to be used for several decades. Subsequent histological studies did not show classic inflammation of muscle or other local tissues as a characteristic of fibromyalgia [19,20]. Other studies on muscle metabolism, when patients with equal general fitness and muscle disuse are used as controls, did not show changes specific to fibromyalgia [20,21]. However, abnormalities in muscle physiology are observed in fibromyalgia [22], with augmented muscle membrane propagation reactions independent of force load or amount of muscle activity, suggesting central deregulation [23]. 

Pain generators within muscles, such as myofascial trigger points, have been shown to modulate generalised tenderness in fibromyalgia [24].

## 4. Neuroinflammation as a Peripheral Pain Mechanism

The 1990 ACR criteria made note of cutaneous dermatographia, related to the release of inflammatory products such as neuropeptides, glutamate, and cytokines, particularly from C-fibre nociceptors in the skin, a process termed neuroinflammation [25]. There are subsequent interactions with both the innate and acquired immune systems and related cells, including keratinocytes and mast cells [26]. Neuroinflammation likely contributes to many of fibromyalgia’s clinical features, such as arthralgia and myalgia, and may account for the increased rate of peripheral neuropathic findings in fibromyalgia [27].

The peripheral C-nociceptors show enhanced spontaneous activity and sensitisation to mechanical stimuli [28] and there is evidence of small nerve fibre pathology in approximately 50% of fibromyalgia patients [27]. These peripheral changes contribute to clinical features including swelling and dysesthesia.

This mechanism also links to other clinical phenotypes that compose the central characteristics of fibromyalgia, such as irritable bowel syndrome, irritable bladder syndrome, migraine, restless legs syndrome (RLS), and multiple chemical sensitivity, among others [29]. The increased activity in C-nociceptive fibre afferents lying behind this process likely relates to central sensitization within the spinal cord’s dorsal horn, as discussed later [26].

## 5. Referred Pain as a Peripheral Pain Mechanism

Building on the work of others [30,31], Smythe suggested that pain from deep spinal structures, such as the lower neck or back, could contribute to the mechanism for the widespread pain distribution [5,32]. These observations continued after the 1990 ACR criteria and remain relevant to a mechanistic understanding of fibromyalgia, but require further exploration in the context of current concepts of central sensitization.

## 6. Characterization of Central Sensitization in Fibromyalgia after the 1990 ACR Criteria

Exploration and understanding of the amplification of sensory inputs to the spinal cord and brain in fibromyalgia accelerated after the 1990 ACR criteria were disseminated. This process, known as central sensitisation, has become recognised as a key mechanism causing a wide range of symptoms in fibromyalgia. The demonstration of a generalised decrease of pain sensitivity in fibromyalgia patients and increased reactivity to peripheral stimulation of nociceptor nerves were important steps to understanding fibromyalgia as a disorder driven by central mechanisms [33,34].

Soon after the 1990 ACR criteria, an early key finding was that A-delta nociceptor stimulation results in increased cerebral evoked responses in the somatosensory cortex [35]. Repetitive stimulation of C-nociceptive fibres results in temporal summation in the spinal cord in normal controls and exaggerates this process in fibromyalgia [36]. Nociceptive-evoked reflex responses in fibromyalgia patients compared to controls showed less peripheral stimulation is required to elicit reflex muscle changes, indicating increased neural sensitivity in the spinal cord [37,38]. These observations indicate increased sensitivity to peripheral nociceptive sensory stimuli in fibromyalgia and reflect the process of central sensitization [39].

The increased excitability of the spinal cord’s dorsal horn neurones is characterised by increased spontaneous neuronal activity, large receptive fields, and augmented stimulus responses, including those transmitted by both large and small calibre primary afferent fibres.

Allodynia, a term that describes pain induced by a non-noxious stimulus, is a key clinical feature of fibromyalgia; this is the mechanism behind abnormal tenderness and relates to increased sensitivity in the large mechanoreceptor fibre group. In the context of central sensitisation, peripheral A-beta fibres, which normally function as mechanoreceptor afferents, interact with sensitised wide dynamic range receptor neurones in the spinal cord’s dorsal horn. The altered neuroplasticity translates innocuous peripheral sensory inputs to pain outputs and provides a link between everyday movements, activities, postures, and other triggers that provoke fibromyalgia pain. This process also has particular relevance to the deeply placed mechanoreceptors in and around spinal structures, such as the lower neck and back. This mechanism would convert mechanoreceptor sensory input to a nociceptor-type function, which results in activation of referred pain mechanisms from the spinal regions, with resultant regionalised pain, tenderness, and other sensory complaints that are typically seen in fibromyalgia. Further evidence to clarify this proposed mechanism is required. 

Even though there is evidence that peripheral nociceptive afferent fibres (i.e., A-delta fibres and C-nociceptor fibres) may play a role in central sensitization [24,40], it is felt that there is little indication of a continuous nociceptive input that would be needed to cause central sensitisation in fibromyalgia. However, the brain’s powerful modulatory effects through descending influences seem to be more important in the fibromyalgia mechanism [41]. 

## 7. Neurotransmitters

In the context of central sensitisation in regards to fibromyalgia, there are a number of neurotransmitters that are elevated compared to controls. These include substance P and glutamate, both of which activate N-methyl-D-aspartate (NMDA) receptors that promote pain transmission [42,43,44].

Substance P, in particular, is a potent neuropeptide released from the terminals of specific sensory nerves and binds to NK-1 receptors. It lowers the synaptic threshold in second-order spinal neurones and, in turn, is released by the activation of NMDA receptors in the dorsal horn. Substance P can travel extensively along the spinal cord to sensitise distant dorsal horn neurones. Substance P is also closely associated with 5-hydroxytryptamine/serotonin (5-HT) in the brain, particularly in areas responsible for emotion and pain perception. Substance P levels are elevated up to three times normal in the cerebrospinal fluid (CSF) of patients with fibromyalgia [45,46,47].

A number of studies also showed increases in glutamate following noxious stimulation in patients with fibromyalgia [48]. These changes are reversed by the administration of the potent NMDA antagonist ketamine in humans [49]. In fibromyalgia, other neuropeptides such as nerve growth factor are also elevated [50] as they are in other painful rheumatic diseases [51]. Other neurotransmitters are also altered to varying degrees, demonstrating the complexity of the pathophysiology. These include calcitonin gene-related peptide, brain-derived neurotrophic factors, corticotrophin-releasing hormone, hemokinen-1, neurokinin A, neurokinin B, adrenomedullin, vasoactive intestinal peptide, neuropeptide Y, and gastrin-releasing peptide [26].

## 8. Descending Pathways in Fibromyalgia

Key brain-to-spinal cord connections originate in the emotion-linked brain regions and pass through mid-brain structures, including the raphe nuclei (upper medulla), the periaqueductal grey, and the locus coeruleus, and then link down to the dorsal horn through reticulospinal fibres. These powerful signaling pathways link supraspinal structures to the activities of the spinal cord sensory transmission neurons. Where these pathways initiate anti-nociceptive activity, the term “diffuse noxious inhibitory control (DNIC) pathway” is used. Dysfunction of this pathway was identified as a fundamental mechanism contributing to pain and other clinical features of fibromyalgia.

These descending pathways involve the monoamine neurotransmitters, 5-HT, and norepinephrine (NE), which modulate the descending inhibitory “tone” that affects transmission neurones associated with dorsal horn pain, and appears important in the facilitation of the pain sensitization process at that level [52,53]. Where there is pain sensitisation in the dorsal horn of the spinal cord through lowered DNIC tone, there is an inability to inhibit transmission of pain-related sensory stimuli, which are then perceived as pain.

Descending pain inhibition is demonstrated in humans by the application of a tonic conditioning nociceptive stimulus. Pain inhibition involving the DNIC is elicited by applying a cold pressor test involving, for instance, submerging the patient’s arm in ice-cold water. In healthy patients, DNIC is demonstrated by the reduction in the patient perception of the initial painful test stimulus at another site. Over time, particularly in the 2000s, several studies showed fibromyalgia patients to demonstrate a lower thermal pain threshold and a lower reduction in the perception of the initial test stimulus after application of the cold pressor test. This indicates that the DNIC is not functioning normally in fibromyalgia [53,54,55]. This process may also involve attenuation of normal “wind-up” pain by C-nociceptive fibre activation in fibromyalgia. Notably, DNIC dysfunction does not occur in depression, highlighting the presence of fundamentally different mechanisms in depression and fibromyalgia [56,57].

The rostral anterior cingulate cortex (rACC) plays a vital role in descending modulatory pain function. Notably, there is an attenuation of rACC function in fibromyalgia. The cerebral response to individually calibrated pain provocation of a pain-free body region, measured by functional magnetic resonance imaging (fMRI), shows higher sensitivity to pain provocation in fibromyalgia patients than in controls. These studies do not show any difference in the activity of these brain regions relating to affect or regions with sensory projections from the stimulated body area. However, fibromyalgia patients failed to respond to pain provocation in the rACC descending pain regulatory system, indicating dysfunction in the downward inhibitory tone from this pathway onto the dorsal horn [58].

NE and 5-HT are the key neurotransmitters of the DNIC pathway. In fibromyalgia, multiple studies showed a reduction of both serum and cerebrospinal fluid concentrations of serotonergic and NE metabolites [42,59,60]. Medications that target and modulate these monoamine neurotransmitters were beneficial in reducing symptoms in some patients in clinical trials [61].

## 9. The Brain in Fibromyalgia

To better define fibromyalgia patients, the 1990 ACR criteria corresponded with developments in neuroimaging; this has subsequently allowed for an enhanced understanding of the neurobiological processes involved in fibromyalgia mechanisms [62]. Several studies showed that there are significant differences in functional neuroimaging in fibromyalgia patients than controls. These relate to central pain processing, differences in affective processing of pain, and modulation of the brain’s influence on spinal cord sensory control mechanisms. 

Single-photon emission computed tomography (SPECT) techniques using radiotracers infer neural activity from localised increases in regional cerebral blood flow (rCBF). A range of abnormalities involving rCBF occurs in fibromyalgia. These abnormalities include reduced flow in the dorsolateral frontal cortical areas of both hemispheres, the thalamus, the head of caudate nucleus, the inferior pontine tegmentum, the superior parietal cortex, and the gyrus rectus [63]. These studies indicated that a range of functional abnormalities related to pain processing occurs in fibromyalgia, and these involve a variety of areas in the brain. SPECT studies also showed hyperperfusion of the somatosensory cortex and related area change. In contrast, hypoperfusion of the amygdala and the anterior insula are significant in the attention dimensions of pain response [64]. There are differences between the findings in these structures between fibromyalgia and depression.

fMRI also demonstrates central neural activation patterns showing increased blood flow to pain processing areas at a lower stimulation threshold in fibromyalgia than in controls [65]. Changes were reported in intrinsic connectivity in fibromyalgia patients compared to controls. The maintenance of the brain’s resting state displays greater connectivity to regions involved in pain processing in fibromyalgia patients than controls [66]. These changes reduce as fibromyalgia pain decreases [67]. Connectivity between the default mode network and pain inhibitory centres is decreased, while connectivity is increased with the insula [68].

Magnetic resonance spectroscopy (MRS), which assesses brain metabolism by determining the concentration of specific metabolites such as glutamate and glutamine, shows fibromyalgia patients have significantly high levels of these compounds in the right posterior insular area compared to controls [69]. This concentration correlates with lower pressure pain thresholds indicating a potential link between these two observations. The alpha-2/delta subunit of voltage-gated calcium channels in pain-related neurons is down-regulated by drugs such as gabapentin or pregabalin, resulting in decreased excitatory release substances, including glutamate and glutamine. Medications that target glutamatergic mechanisms, such as these alpha-2/delta ligands, may be beneficial in fibromyalgia [61].

In the last decade, understanding of glial cell activation associated with neuroinflammation has increased. This process is inferred by the elevation of cytokine IL-8, but not IL-1β in the CSF of fibromyalgia patients compared to controls [70,71]. IL-8 is co-localised with the translocator protein (TSPO) in glial cells, which is the rate-limiting step in serotonin synthesis, and hence, it modulates serotonergic synaptic transmission, and descending pain modulation. In fibromyalgia, genetic polymorphisms of TSPO are associated with symptom severity, cerebral pain processing, and interact with the serotonin transporter gene [71]. Brain glial activation, as seen on PET scans, show widespread cortical activations in fibromyalgia patients compared to controls, and correlates with fatigue [72]. MRS techniques show neuroinflammation in fibromyalgia [73].

## 10. Genetic Factors

Since the 1990 ACR criteria allowed better classification of fibromyalgia, further research of affected families and twin studies showed that up to 50% may be genetic factors [74]. Genetic factors modulate activity in relevant neurobiological systems, such as stress-response systems [75,76,77]. Further understanding of the relationship of genetic factors to fibromyalgia phenotypes, such as early onset fibromyalgia, may allow for different management strategies in different subsets [78].

## 11. Psychological Factors in Fibromyalgia

Several psychological factors may be relevant to the central processes causing pain in fibromyalgia patients [57]. These factors were studied and reviewed in the decade after the 1990 ACR criteria [79]. Patients with fibromyalgia often react adversely to a psychological input that is perceived to be stressful [80]. Some people are more prone to this abnormal stress reactivity than others. Patients with fibromyalgia are more likely to have personalities characterised as neurotic, defined as an enduring tendency to experience negative emotional states, using routine personality classification [81]. This type of personality is more prone to react to stress adversely. Other relevant psychological inputs include poor coping abilities and tendencies to calamity under stressful situations [82]. These types of processes often overlap. The subsequent stress reaction links to the processes modulating the downward pain control centers from the brain and mid-brain to the dorsal horn [83].

Persons with fibromyalgia tend to be more anxious, with increased chances of depression compared to controls [84]. The lifetime rate of depression in persons with fibromyalgia may be up to approximately 50% to 60%, and the point prevalence is around 20% to 25%. The mechanisms of depression include changes in similar monoamine transmitters, such as serotonin and NE, as occur in fibromyalgia. Some medications that target fibromyalgia pain also target depression [61]. However, other medications, such as selective serotonin reuptake inhibitors, significantly help depression but may not modify fibromyalgia pain.

Depression does not cause fibromyalgia; hence, it is a common comorbid factor rather than a causative factor.

## 12. Sleep in Fibromyalgia

Early studies by Moldofsky [85] suggested that sleep disturbance might precede the onset and contribute to symptoms of fibromyalgia. Understanding the importance of sleep in fibromyalgia preceded the 1990 ACR criteria, but has been clarified in decades since. For instance, sleep deprivation was shown to impair descending pain modulation pathways important in pain control and coping with pain [86].

## 13. Stress Reactivity in Fibromyalgia

The hypothalamic–pituitary–adrenal (HPA) axis links psychological and emotional factors to neuroendocrine output. Many studies explored the role of this stress axis in a variety of chronic pain conditions, including fibromyalgia [87,88]. Dysfunction occurs in various elements of the HPA axis, with elevated basal levels of adrenocorticotropic hormone (ACTH) and abnormal secretion in response to stress. Patients also have lower levels of growth hormone, insulin-like growth factor-1, thyroxin, estrogen, and urinary cortisol [89].

It has been postulated that some changes in neuroendocrine function in fibromyalgia patients may contribute to some symptoms contributing to a characteristic phenotype, such as fatigue. Blind studies replacing growth hormone reported improvement in many of the characteristic symptoms, such as tenderness, and overall well-being [90]. Many other neuropeptides, including neuropeptide Y, are also altered in fibromyalgia patients compared to controls, but their clinical significance is unclear [91].

Evaluation of the sympathetic nervous system through measurement of heart rate variability shows excessive sympathetic tone and sympathetic reactivity to stress [92].

## 14. Social Factors

A range of psychosocial factors has been linked to onset, exacerbation, or perpetuation of fibromyalgia [57,93]. Illness burden and emotional distress are highly associated with fibromyalgia, likely related to neurophysiological consequences of activation of the stress response [94,95]. These are not the subject of this review.

## 15. Summary

Understanding of mechanisms contributing to the fibromyalgia phenotype has evolved with considerable benefit derived from the promulgation of the 1990 ACR criteria. Mechanisms relevant to fibromyalgia are grounded in increased knowledge of the interaction between stress-response systems and sensory modulation, with a particular interest in pain-related neural functioning. 

Since the 1990 ACR classification criteria, the criteria have evolved so that fibromyalgia features are seen as occurring on a spectrum [9]. This development reflects the variable nature of psychosocial inputs and neurophysiological responses linked to fibromyalgia’s clinical features.

## Figures and Tables

**Table 1:** Key mechanisms underlying fibromyalgia advanced by ACR 1990 criteria.

Neural Mechanisms	Type of Mechanism
Peripheral	Mechanoreceptor input
	Referred pain
	Nociception
	Sympathetic nervous system
	Neuroinflammation
Spinal cord	Central sensitization
	Descending spinal cord control
Brain	Neurotransmitter changes
	Connectivity changes
	Neuroinflammation
**Other mechanisms**	Genetic
	Psychological
	Stress reactivity
	Social factors
